# Sol‐Gel‐Syntheses and Structural as well as Electrical Characterizations of Anatase‐ and Rutile‐Type Solid Solutions in the System IrO_2_−TiO_2_

**DOI:** 10.1002/open.202300032

**Published:** 2023-07-19

**Authors:** Daniel Reichert, Klaus Stöwe

**Affiliations:** ^1^ Institute of Chemistry Faculty of Natural Sciences Chemnitz University of Technology Straße der Nationen 62 09111 Chemnitz Germany

**Keywords:** conductivity measurements, mixed metal oxides, Rietveld refinement, sol-gel syntheses, X-ray diffraction

## Abstract

This paper describes solid solutions in the quasibinary oxide system iridium‐titanium IrO_2_−TiO_2_ with rutile and anatase crystal structures. Based on X‐ray diffraction evaluations using Rietveld refinements, changes of lattice parameters were determined within the composition series of 0–100 mol % iridium. These changes prove the existence of a complete solid solution series in the rutile structure type. The solubility limit for iridium in the anatase lattice was found to be 6.0(8) mol % iridium for the underlying sol‐gel process. In addition, iridium is a promoter for the conversion from anatase to rutile type. Furthermore, the X‐ray diffraction results of a calcination temperature series for the composition with 5 mol % iridium are shown, which confirm the findings of the composition series and allow conclusions on the phase segregation behavior. The results are complemented by 2‐point conductivity measurements at different pressures in a piston press to investigate the question of the conductivity mechanism.

## Graphical abstract

Solid solutions in the IrO_2_−TiO_2_ quasibinary iridium‐titanium system with rutile and anatase crystal structures were observed for the first time based on powder X‐ray diffraction measurements. Evaluations using Rietveld refinements showed the existence of a complete solid‐solution series in the rutile structure type. Conductivity measurements using the 2‐point method support these results by clearly distinguishing the data from those of the percolation theory.

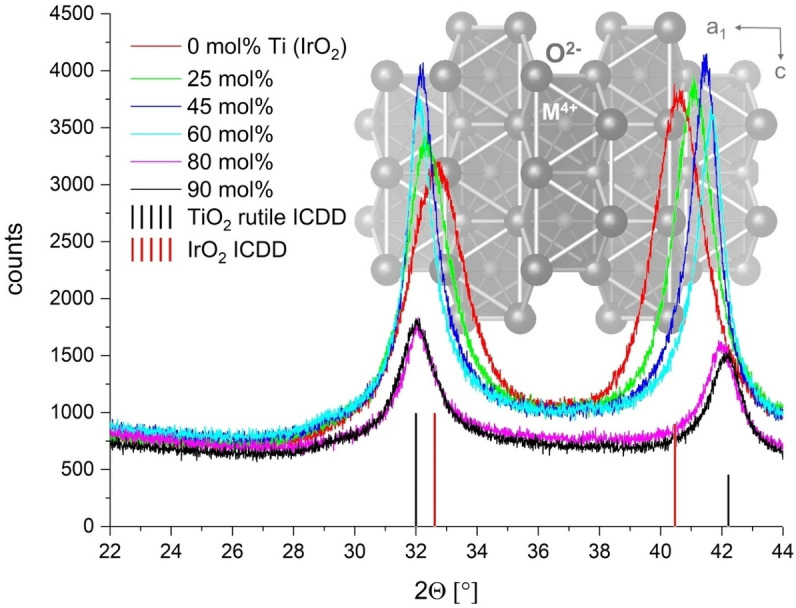

## Introduction

Mixed oxide systems consisting of different binary or ternary metal combinations are of great scientific and technical interest. During the synthesis process, substances can be formed which differ significantly in their properties from their respective pure counterparts.^[1]^ In addition, certain properties of very expensive noble metals or metal oxides can be retained by using inexpensive matrix metal oxides with very little material input. In materials science, mathematical theories such as percolation theory^[2]^ are used to explain the behavior, according to which only a certain amount of dopant is required to imprint the properties, such as metallic conductivity, of a dopant into the matrix component.^[3]^ Thus, in some cases, intrinsically electrocatalytically inactive substances may be suitable for use in electrolyzers or fuel cells.^[4]^ Besides this, other applications of doped titanium dioxides are often described as variants for pollutant degradation that show enhanced activity when irradiated with visible light.^[5]^

The targeted synthesis of mixed oxides is non‐trivial in many cases and requires a great deal of research effort, since, in many cases, numerous influencing parameters and target variables have to be taken into account. Both the composition of a formulation and the synthesis process determine the properties of the resulting products. Especially in the frequently used sol‐gel process, there is a complex relationship between possible synthesis parameters and target functions such as conductivity or catalytic activity.^[6]^ For example, process parameters such as the type and quality of the inert gas used, the type of reactor or the calcination conditions may play a role. On the other hand, material parameters such as the choice of metal precursors, solvents or even the type of stabilization by complexation with respect to hydrolysis rates can have an impact on the specific surface area of the produced powders. The last point plays an enormous role, especially when metal alkoxides are used as precursors. If the different hydrolysis rates are poorly coordinated, the individual metals may not be present in an atomic level distribution in the gel, but domains of separate hydrolysates may form. Thus, even at moderate calcination end temperatures, solid solutions cannot be obtained because the interdiffusion of the separated phases is limited in a solid state reaction. The resulting product then corresponds more to a metal oxide mixture than to a mixed oxide.

In this work, the oxide system iridium‐titanium is subjected to a detailed investigation. Titanium dioxide crystallizes, among others, in the two metastable modifications anatase and brookite and the thermodynamically stable modification rutile.^[7]^ Only one rutile type modification stable at room temperature and ambient pressure is known for iridium dioxide.^[8]^ The current state of the scientific literature suggests that no solid solutions of the anatase structure type are known or possible in the IrO_2_−TiO_2_ system. Since both binary oxides are known in the same rutile structure type, solid solution formation between the two is expected without gaps. However, there are not many sources where solid solution occurrence has been observed or comprehensively demonstrated. In one of the earliest publications on this subject by McDaniel and Schneider,^[9]^ solid‐state reactions of the binary oxides were performed over the entire compositional range, and the authors concluded that solid solutions did not exist in the system. Later it was found^[10]^ that in the presence of iridium the anatase phase of TiO_2_ is completely suppressed and only a rutile phase occurs instead. A shift in the X‐ray diffraction reflection positions due to a doping effect has not been proven by the results of the paper. It was not until 2002 that the group around Endo^[11]^ succeeded in demonstrating solid solution formation by means of lattice parameter changes. Up to an Ir content of 50 mol %, a complete solid solution series was observed. At higher Ir contents, a second rutile phase of pure iridium dioxide appeared. In 2007 and 2008, two publications[^12^, ^13^] by Crayston's group appeared. The team had used iridium precursors containing chlorine and sodium to prepare the samples.^[12]^ Despite washing after calcination, it cannot be ruled out that the lattice deformations are caused by chloride ions embedded in the lattice. Quantitative determinations of the chlorine content in our own investigations (see below) confirmed the finding of residual chlorine content at a calcination end temperature of 400 °C. In addition, Crayston^[12]^ showed, in 2007, that mixtures of anatase−TiO_2_, rutile−IrO_2_ and a solid solution phase were present in the specimens. In another work,^[13]^ the use of chlorine‐ and sodium‐containing starting materials in the sol‐gel process was omitted. Here, an iridium acetate precursor was used in a water‐rich and an ethanol‐rich route. All samples were free of phases with anatase structure. Only one rutile phase was found. The authors published only the change in the lattice parameter *a* and concluded a continuous solid solution series. The results of Cruz's group,^[14]^ who also used precursor‐based methods, are another important reference for the findings in this work. The study of thin films resulted in anatase−rutile mixtures where iridium dioxide was segregated from the surface of the film. In 2014, the research group of Ohno^[15]^ succeeded in synthesizing a solid solution phase with brookite structure. The authors give a solubility limit of 0.5 wt % Ir. Limited Ir solubility also appears to be present in the metastable phases of titanium dioxide. Oakton et al. developed a synthesis protocol for the preparation of chlorine‐free IrO_2_ nanoparticles dispersed in TiO_2_ with high total surface area^[16]^ based on the Adam's melting method. Their objective was to reduce the cost of catalysts in the application of oxygen evolution reaction (OER). In interpreting the powder diffraction patterns of the products, they admit that the similarity of the two crystal structures in the rutile type as well as the X‐ray diffraction reflection half‐widths make further interpretation difficult. However, elemental mapping by energy dispersive X‐ray spectroscopy (EDXS) revealed iridium‐rich regions in the samples. IrO_2_/TiO_2_ superlattices fabricated by molecular beam epitaxy (MBE) are described by Kawasaki et al.,^[17]^ where the stresses generated by the different lattice parameters of the two phases are compensated by ordered interphase reconstructions. The superlattices retain their metallic conductivity down to three atomic layers. IrO_2_−IrO_2_ subband couplings were found using angle‐resolved photoemission spectroscopy (AR‐PES) measurements. In 2020, hypothetical anatase type TiO_2_−IrO_2_ solid solutions as prospective electrocatalysts for OER have been modelled regarding their theoretical overpotentials of surface Ir sites. The conclusion was that the anatase crystal phase TiO_2_−IrO_2_ solid solution is of high importance for bringing about highly active Ir sites for OER.^[18]^ Except as electrolysis catalysts, solid solutions in the system IrO_2_−TiO_2_ might also find application as electrocatalysts in fuel cells. IrO_2_ is known for its outstanding chemical stability coupled with high electrical conductivity. However, the high price of the noble metal Ir limits its general application. Therefore, the contribution also aims to determine the limits up to which “dilution” with semiconducting TiO_2_ is possible in the IrO_2_−TiO_2_ system.

## Results and Discussion

### Characterization by X‐ray powder diffraction

#### Lattice parameter changes

The powder samples obtained were characterized by powder X‐ray diffraction (PXRD). In the absence of iridium, the TiO_2_ anatase modification forms without any other crystalline phase impurities. Anatase–rutile mixtures are formed at small Ir doping levels. Only above a doping level of ca. 10 mol % Ir, the anatase phase is no longer detectable. Figure SI1 shows parts of some diffraction pattern and illustrates the shift of the reflections (110) and (011) of the rutile phases due to Ir doping.

**Figure 1:**
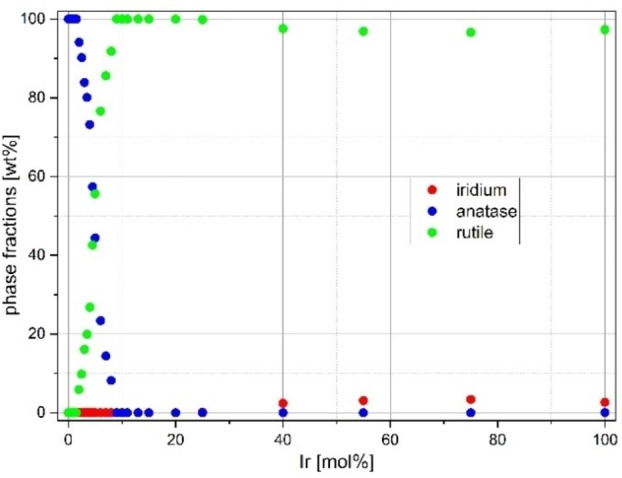
Phase fractions of the doping series Ir_x_Ti_1−x_O_2_ synthesized via a modified Pechini sol‐gel route after calcination at *T*=400 °C.

This shift in the reflection position is also observed for the TiO_2_−anatase phase, but to a much lesser extent. These effects can already be interpreted as an indication of complete solid solution formation, since the reflection positions of the two pure rutile phases of TiO_2_ and IrO_2_ converge over the entire doping range. Moreover, there are no clear indications of superstructure reflections, reflection splittings or superpositions of reflections of several different phases (cf. chapter “Refinement with two rutile phases”). A statistical substitution of Ir and Ti on the cation sites is assumed. Figure 1 shows the crystalline phase composition of all prepared substances of the doping series obtained by Rietveld refinement.

It can be seen that with increasing iridium content the anatase phase fraction decreases very rapidly and disappears completely at about 8–10 mol % Ir. It is conceivable that the iridium incorporation favors the formation of a rutile modification. For comparison, we refer to the literature (Ref. [19]), where rutile promoters and inhibitors are listed, but iridium was not considered. In addition, at high Ir concentrations, low intensity reflections can be assigned to metallic iridium in the PXRDs, but they have a very small full width at half maximum (FWHM) and therefore can be well fitted and evaluated in the Rietveld refinement. Iridium metallic precipitates are observed due to the disproportionation of the trivalent precursor into IrO_2_ and Ir as a kinetic effect, as well as the incomplete oxidation of the Ir metal during calcination.

**Figure 2:**
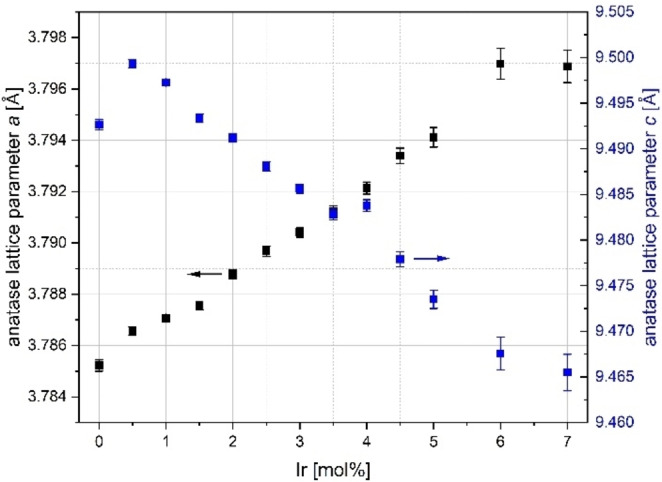
Lattice parameter changes of solid solutions with anatase type crystal structure of the doping series Ir_x_Ti_1−x_O_2_ synthesized via a modified Pechini sol‐gel route after calcination at *T*=400 °C; above 7 mol % Ir the reflection intensities of the anatase phase are too weak to be evaluated quantitatively.

A clear indication of solid solution formation in the two oxide phases is the observation of the lattice parameter changes over the studied composition range. Figure 2 shows the corresponding plot for the anatase phase.

For an accurate evaluation of a phase, a sufficiently high fraction should be present. Therefore values were only considered if at least 10 wt. % of the considered phase were present.

There is a continuous change of the lattice parameter of the tetragonal unit cell in the space group *I*4_1_ */amd* (No. 141). The lattice parameter *a* of the undoped TiO_2_ anatase phase is reported to be *a*=3.785 Å and *c*=9.514 Å.^[20]^ The values obtained in this work by Rietveld refinement fit relatively well with the literature values,^[21]^ except for the anomaly in the parameter *c* (see Figure 2 at 0 mol % Ir). Based on incomplete thermolysis (from thermal gravimetric analysis / differential scanning calorimetry (TGA/DSC) data up to final calcination temperature), it is assumed that the reduction of the lattice parameter is caused by carbon impurities. Iridium‐containing samples appear to promote carbon removal during calcination, so no anomalies occur here. It should be noted that the error bars of the Rietveld refinement standard deviations are usually smaller than the symbol size in Figure 2. The errors correlate directly with crystallite size and phase fraction, especially in case of superpositions with reflections of other mixture components.

The crystallite sizes for the individual samples can be found in the Supporting Information (see Tables SI2–3, all other parameters Table SI1). In the Rietveld refinements,^[20]^ the z(O) positional parameter of the crystallographic position O (8e) 0 0 z (M=Ir, Ti at (4a) 0 0 0) is also freely refinable. Figure SI2 shows the obtained values of z(O) and the cell volume (see also Table SI2). The observed changes of the lattice parameters, the cell volume and the oxygen position parameter are clear indications that substitutional solid solution formation Ir↔Ti is present in the anatase structure type. For a determination of the partial site occupation by the Rietveld refinement program *Topas*, the presence of a substitutional solid solution was modelled by refinement of the site occupation parameter (sof) of the cation sites. The solubility limit can be given by the site occupation with 6.0(8) at % Ir at a nominal doping degree of 7 mol %.

Furthermore, we refer to Figure SI4 (top), in which the Ir site occupation factors in the two structural polymorphs are compared.

**Figure 3:**
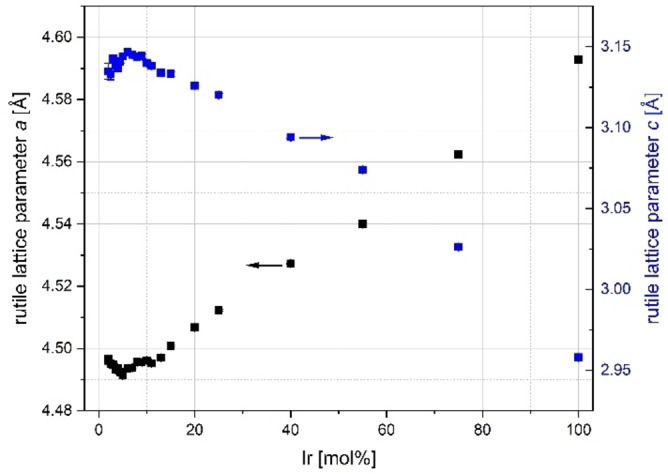
Lattice parameter changes of solid solutions with rutile type crystal structure of the doping series Ir_x_Ti_1−x_O_2_ synthesized via a modified Pechini sol‐gel route after calcination at *T*=400 °C.

Figure 3 shows the change in lattice parameters of all rutile solid solutions prepared.

In solid solutions, the lattice parameters usually change in such a way that the crystallographic parameters of the two pure phases (with the same lattice type) approach each other (Vegard's rule).^[21]^ In this case, it means that the lattice parameter *a* increases and the lattice parameter *c* decreases (see also Table SI1). The same happens to the volumes of the unit cells, which continuously approach. Both lattice parameters *a* and *c* of the tetragonal unit cell with the space group *P*4_2_/*mnm* (No. 136) remain approximately constant at small nominal doping degrees up to approx. 7 mol % Ir. Further interpretation is very difficult because of the low mass fractions and the small crystallite sizes, the associated high reflection widths and low intensities as well as the resulting large positional deviations (cf. with anatase). At higher doping degrees, the expected behavior becomes apparent. The values of the lattice parameters for the pure iridium dioxide phase of *a*=4.4971(3) Å and *c*=3.1420(3) Å are slightly below the literature values with *a*=4.5051(3) Å and *c*=3.1586(2) Å.^[22]^ Literature values of *a*=4.594(3) Å and *c*=2.959(2) Å were found for the lattice parameters of the pure titanium oxide phase.^[23]^ It should be noted that the literature values vary from publication to publication and that the cell parameters are influenced by the synthesis route to an extent that should not be underestimated (influence of the chlorine, sulfur, nitrogen and oxygen content in the oxide).

#### Positional and occupational parameter changes

**Figure 4:**
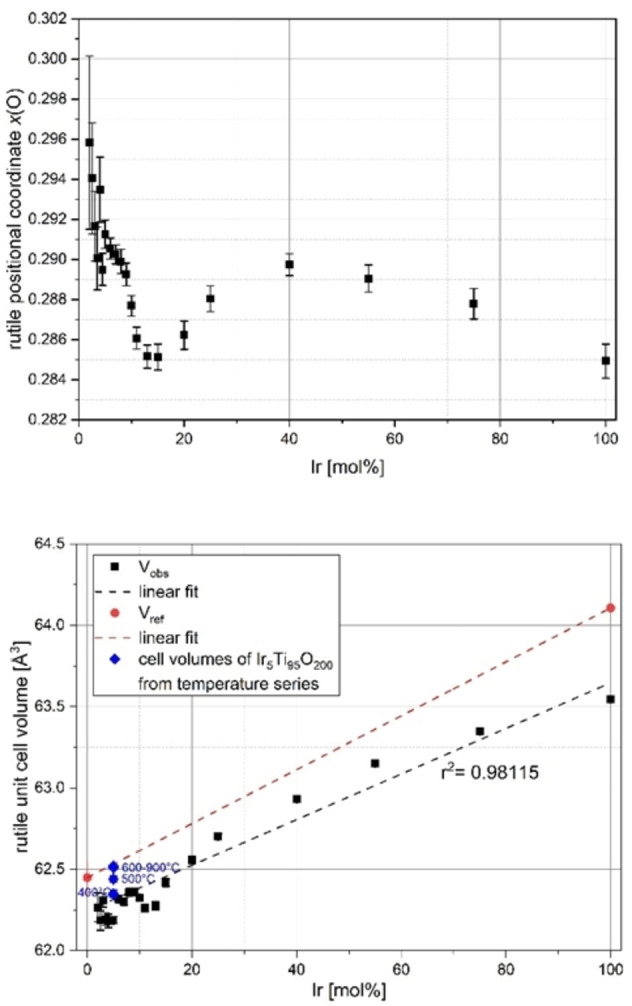
Change of the positional parameter of the oxygen atoms x(O) (top) and the unit cell volume V_obs_ (bottom) in the rutile structure type in the doping series Ir_x_Ti_1−x_O_2_ synthesized via a modified Pechini sol‐gel route after calcination at *T*=400 °C; reference data V_ref_ were calculated from lattice parameters specified in Table SI1; values as blue diamonds were taken from temperature series of Ir_0.05_Ti_0.95_O_2_ (calcination temperatures 400–900 °C).

The positional parameter *x* for the oxygen atoms, x(O), was released during the refinement of the crystallographic parameters with *Topas*. Figure 4 gives an overview of the values obtained for the solid solutions of the rutile structure type (see also Table SI3). The obtained cell volumes are represented, too. As shown by the inclusion of data from the temperature series of Ir_0.05_Ti_0.95_O_2_ (calcination temperatures from 400 °C to 900 °C; blue diamonds in Figure 4), for the two boundary phases the differences in the observed unit cell volumes compared to those calculated from literature data are due to variations in the thermal history of the samples. Overall, the values of the position parameters x(O) can be divided into two regions separated by the polymorphic anatase‐rutile phase transition in the system under consideration. With the free parameters *a*, *c* and *x*(O) in the space group *P*4_2_ */mnm* (No. 136), the rutile structure is crystallographically fully described. The rutile structure is derived from the hexagonal densest sphere packing with a half occupation of the octahedral voids. The planarization of the trigonal pyramidal Ti coordination around the O atoms leads to a tetragonal lattice type in which the cation coordination polyhedra are connected by common edges and corners. Due to the connection of the rectangular bipyramids (^[4+2]^ coordination) with the point symmetry *mmm* (despite this symmetry commonly called “octahedron”) via common edges to strands in *c* direction and the resulting relatively short metal–metal distances, characteristic structural and electrical properties arise in the dioxides of the transition elements. Due to additional d electrons of the elements following Ti in the periodic system of elements (PSE) as, for example, V, Cr, Mo etc., the dioxides show the behavior of so‐called Peierls distortions. Only in the electron‐rich dioxides of Ru and Ir, the undistorted rutile structural type is observed again.^[24]^ At about 15 mol % Ir, Figure 4 (top) shows a local minimum of *x(O*) at an x‐value comparable to IrO_2_. With increasing iridium content, geometrical changes occur in the rutile structure due to the different ionic radii of Ti^4+^ (r=60.6 pm for CN=6) and Ir^4+^ (r=62.5 pm for CN=6^[25]^), as well as electronic changes due to the different valence electron configurations. The cell volumes of the solid solution series follow a straight line, as expected according to Vegard's rule, but all experimental values are slightly lower than the literature data.

**Figure 5:**
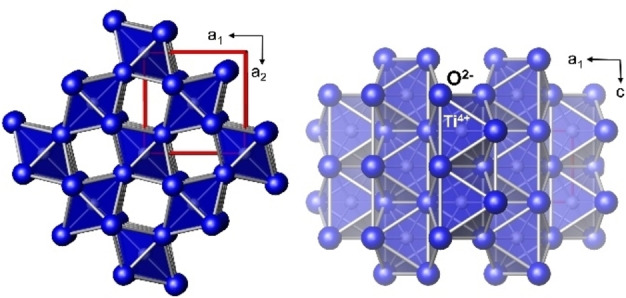
Crystal structure of the rutile type TiO_2_; left: projection down direction [001] right: projection down [010].

Figure 5 shows the crystal structure of the rutile type with the connectivity of the cation coordination polyhedra. When calculating the interatomic distances using the refinement results with *Topas*, a transition from a slightly compressed bipyramid (TiO_2_‐like coordination) to a significantly compressed bipyramid (IrO_2_) is observed, assuming pure TiO_2_ and increasing the Ir doping content.

Figure SI3 shows the refinement results of the substitution occupation in the cation sublattices of the anatase and rutile structure types in form of site occupation factors (sof) of Ir.

In both titanium dioxide phases, iridium contents on the cation positions of the two structure types are shown as refinement results. The iridium content increases continuously with the degree of doping, which is a good indication of complete solid solution formation. However, for the anatase type, the site occupation is somewhat lower than the nominal doping level would suggest. The expected values are very valid for the rutile phase. However, the presence of amorphous fractions, which cannot be detected by PXRD, cannot be excluded.

### PXRD pattern refinement with two rutile phases

No reflection splittings were observed in the measured diffraction pattern, which could have been interpreted as evidence for the presence of a phase mixture of two IrO_2_ and TiO_2_‐rich rutile phases. However, theoretically, in the presence of a mixture of a Ti‐rich and an Ir‐rich rutile solid solution, the differences in the lattice parameters of the two mixed phases become smaller and smaller as the metal contents in the boundary phases coincide, until in the limiting case of equal contents of both metals only a single solid solution phase is present. In the case of local segregation below the coherence length of the X‐ray beam, the transition from a two‐phase to a single‐phase system could also be continuous.

Therefore, for the large half‐widths of the reflections, one refinement model with two rutile phases with released Ir occupation components was adapted as an alternative and the results of both refinement models were compared. Figure SI4 shows a comparison of the two refinement models, model 1 and model 2, see Table SI4, of a diffraction pattern of a sample with a nominal doping degree of 40 mol % Ir. However, there is no clear difference in the curves between measured and fitted values. A data refinement always improves when more refinement parameters are released, but the question is whether the improvement in refinement quality with more parameters is statistically significant or not. In the present case, the second rutile phase increased the number of free parameters by six (*a*, *c*, scale, L, *x*(O), and *sof*(Ir)), but the refinement quality decreased only by about 0.9 %. All in all, we tend to assume a single solid solution phase in the rutile structure type. Nevertheless, it cannot be excluded that the powder samples show some inhomogeneities in terms of iridium‐ and titanium‐rich domains. Answering this question is beyond the capabilities of powder X‐ray diffraction, but additional clues can be obtained from observing the change in diffraction patterns when the samples are calcined at higher temperatures.

### Consideration of the thermal stability of the solid solution obtained for composition Ir_0.05_Ti_0.95_O_2_

#### Phase fractions and crystallite size

To improve the understanding of solid solution formation in the synthesized compounds and the evaluation by Rietveld refinements, diffraction experiments were performed to clarify the temperature dependent behavior (results see also Table SI5, diffraction pattern changes in Figure SI5). Higher calcination end temperatures change crystal structure parameters, phase fractions and microstructure parameters of the powders such as crystallite size. With increasing crystallinity, the FWHM of the reflections become smaller, so that clearer conclusions about phase compositions can be made for highly superimposed reflection intensities.

However, as the calcination temperature increases, many phase diagrams also account for the segregation of solid solutions into their boundary phases. A good example of this is the RuO_2_−TiO_2_ system:^[26]^ at temperatures above *T*=1227 °C, the system separates into RuO_2_‐rich and TiO_2_‐rich solid solutions with decreasing limiting solubilities, finally decomposing into Ru metal‐rich and TiO_2_‐rich solid solutions at *T*=1425 °C. Therefore, the temperature behavior is of limited importance for the stability of solid solutions at lower temperatures. However, the lattice parameters can be determined with higher accuracy. The required samples were first obtained by parallel synthesis at an iridium content of 5 mol % and all samples were calcined at *T*=400 °C. Subsequently, the corresponding samples of each temperature level were removed from the cooled furnace and the remaining samples were heated to the next higher final calcination temperature. This process was repeated up to *T*=900 °C in 100 °C steps.

**Figure 6:**
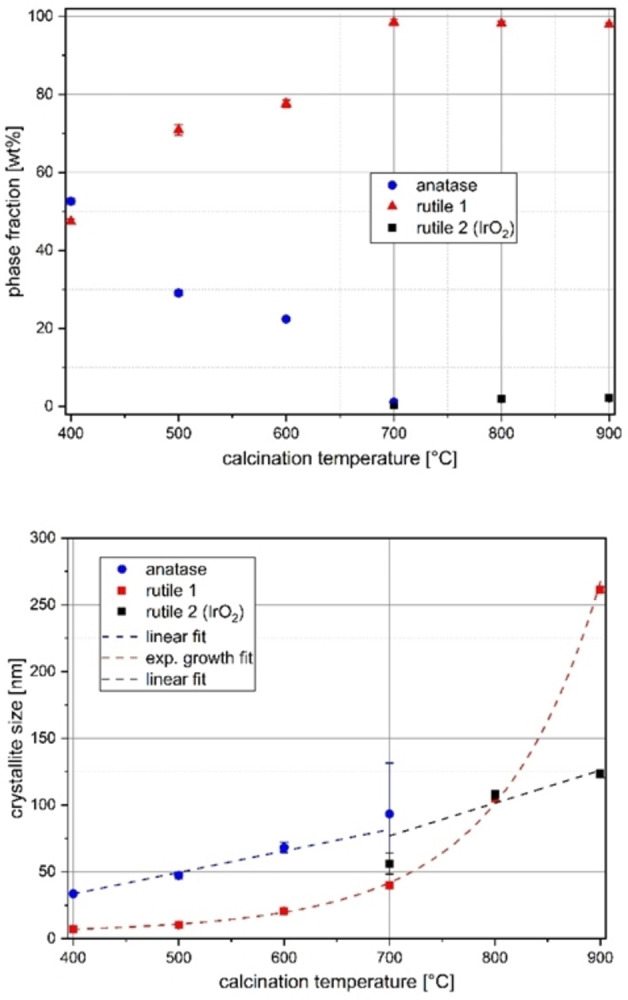
Temperature dependence of phase fractions (top) and crystallite sizes (bottom) of Ir_0.05_Ti_0.95_O_2_ samples prepared via a modified Pechini sol‐gel route after calcination at different temperatures. Dashed fit curves for eye guidance.

Figure 6 shows the change in both crystallite sizes and phase fractions with increasing temperature. The upper part of Figure 6 shows the expected decrease of the phase fraction in the metastable anatase phase. At a temperature of *T*=600 °C, reflections of a second rutile phase, IrO_2_, are visible, but these could only be refined by defining the crystallite size and position parameters *x*(O), and were therefore not included in the Figures (phase fraction below 0.3 %). Even at higher temperatures, the phase fraction of IrO_2_ remains very low. Since the reflections at higher calcination temperatures have a small FWHM, they can be very well evaluated in the refinement procedure.

#### Lattice parameter changes

**Figure 7:**
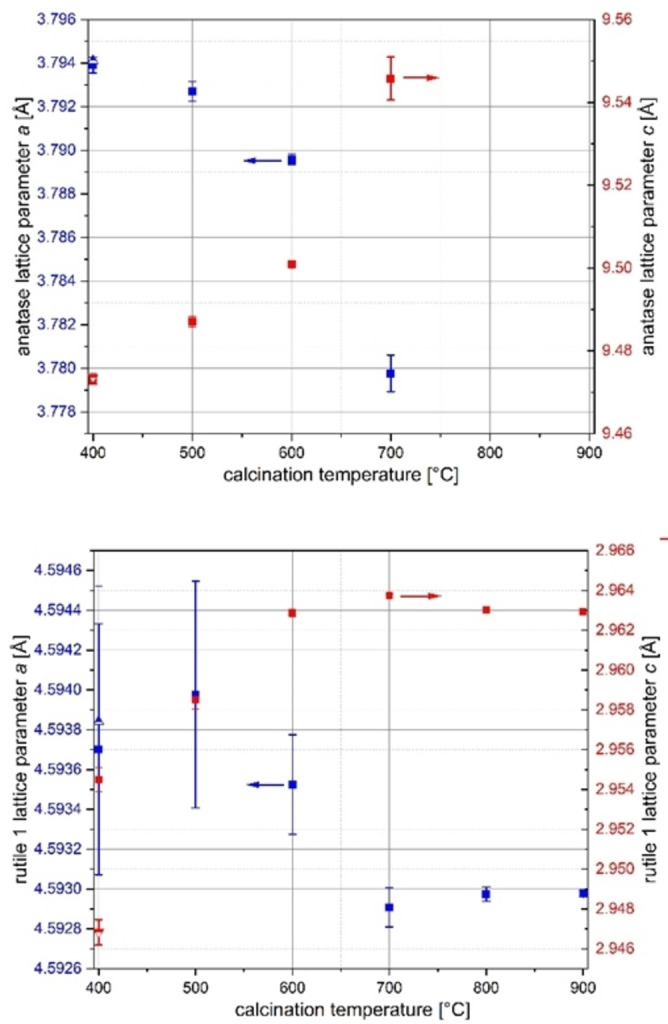
Temperature dependence of the lattice parameters of the anatase (top) and rutile 1 phase (bottom) in samples of the composition Ir_0.05_Ti_0.95_O_2_ prepared via a modified Pechini sol‐gel route after calcination at different temperatures (half‐filled symbols at 400 °C: values from doping series).

Figure 7 shows the lattice parameter changes of the anatase and (titanium‐rich) rutile phases in comparison. With higher calcination temperatures, the crystallinity increases, that is, the reflections become more intense and their FWHM decreases, so that the standard deviations of the individual lattice parameters also decrease. This is particularly evident for the lattice parameter *a* of rutile phase 1 (Figure 7, bottom), where the error bar at lower temperatures is comparatively high relative to the change in the parameter itself. The deviation of the value *c* between the doping series and the temperature series at *T*=400 °C shows the sensitivity of the system to the preparation parameters. For the parameter *a*, the two values are relatively close despite large error bars.

The lattice parameter *a* of the anatase phase decreases (Figure 7, top), while the lattice parameter *c* increases with increasing calcination temperature, that is, it moves toward the values of the undoped boundary phase TiO_2_ in the anatase structure type, compare to Figure 2. It can be concluded that the anatase phase not only decreases in proportion but also exhibits segregation. This segregation is supported by the decreasing site occupation of the cation position with iridium (Figure SI6, top), which reaches a value of zero at *T*=700 °C. To draw a corresponding conclusion from the behavior of the lattice parameters of the rutile phase is not obvious, since for a nominal composition of Ir_0.05_Ti_0.95_O_2_, only a maximum in *a* and a minimum in *c* with a low variability around this point are observed in the lattice parameters of the phase as shown in Figure 3.

Both the presented results of the lattice parameter changes and the site occupation factors at the lowest calcination temperature of *T*=400 °C show good agreement with the values of the doping series (replicate samples).The refined total content of the element Ir in all three phases remains well below the nominal content of 5 mol % at all calcination temperatures (red line in Figure SI6, bottom), but asymptotically approaches the expected value as the calcination temperature increases. At higher temperatures, the sample becomes more crystalline due to the formation of an IrO_2_ phase and more refinable by the Rietveld method.

Overall, the results of the calcination series show that at a nominal composition of Ir_0.05_Ti_0.95_O_2_, the solid solutions formed at a calcination temperature of *T*=400 °C in the TiO_2_−IrO_2_ system transform and segregate exclusively into the rutile structure type when the temperature is increased from an initial approximately 1 : 1 mixture of anatase and rutile, so that a second phase with a rutile structure is observed starting from *T*=700 °C calcination temperature, which was assigned to pure IrO_2_. In addition to segregation, crystallite size growth also occurs, so that as the temperature increases, the refined Ir content also approaches the nominal content.

#### Interatomic distances

The interatomic distances can also be calculated from the crystal structure data and compared with each other (see Table SI6). In the rutile structure type with space group *P*4_2_ */mnm*, the metal ions occupy the Wykoff position *2a* (000) with point symmetry mmm, the oxygen atoms occupy position *4f* (xx0) with point symmetry *m2m*; that is, the coordination polyhedron is a rectangular bipyramid with two different metal‐oxygen distances, axial (2x) and equatorial (4x), and three different O−O distances, the rectangular polyhedral surface with the shared edges to neighboring polyhedra (2x) and the heights corresponding to the lattice parameter c (2x) and finally eight distances of the rectangular surface atoms to the two axial oxygen atoms.

**Figure 8:**
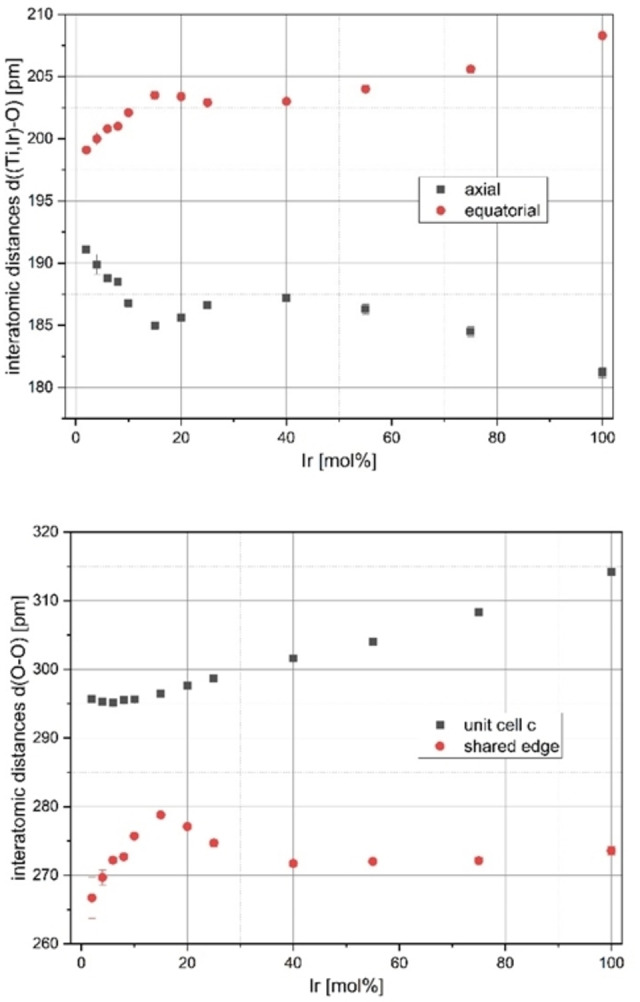
Interatomic distances in the cation coordination polyhedron of the rutile structure type, a rectangular bipyramid (“octahedron”) calculated from crystal structure data refined with *Topas* as a function of the degree of doping; top: axial (2x) and equatorial distances *d*(Ti,Ir)−O (4x) of the composition Ir_x_Ti_1−x_O_2_ produced by a modified Pechini sol‐gel route after calcination at *T*=400 °C; bottom: distances *d*(O−O) in the rectangle of the coordination polyhedra, which are connected to strands along direction [001] by sharing a common edge.

The selected distances are plotted against the degree of Ir doping in Figure 8. The axial and equatorial distances are also frequently considered and discussed in the literature, as in, for example, References [22] and [27]. Figure 8 (top) shows the corresponding distances for the Ir_x_Ti_1−x_O_2_ composition series. With increasing Ir content, there is a transition from a slightly compressed bipyramid (TiO_2_‐like crystals, cf. Ref. [27]) to a significantly compressed bipyramid (IrO_2_,^[22]^). By extrapolating the values on the y axis, it is clear that no stretched bipyramid occurs at 0 mol % Ir. This finding is in contrast to the literature,[^27^, ^28^, ^29^] according to which stretched bipyramids are found for TiO_2_. However, we would like to point out our own results on the temperature dependence of the oxygen position parameters at a doping level of 5 mol % Ir (see Table SI5). At higher temperatures, the *x*(O) resemble the literature results for TiO_2_, for which stretched polyhedra can also be calculated. Thus, the oxygen positional parameters are more dependent on the final calcination temperature than on the iridium content.

Overall, it is clear that the deformation of the cation coordination polyhedron is similar to that of IrO_2_ even at small iridium contents and that, therefore, there must be similarities to the band structure of IrO_2_ with its high conductivity.

The ionic radii of the two metal ions involved differ by only 2 pm (Ti^4+^ 60.5 pm, Ir^4+^ 62.5 pm; for CN=6[^25^, ^29^]). Thus, the changes shown in Figure 8 bottom are not due to size effects in the incorporation of the slightly larger Ir^4+^ cation.

The shown O−O distances occur in the rectangular base of the bipyramid as cation coordination polyhedra, the area of which tends to increase, reducing the repulsion of these oxygen anions. At a doping level of 15 mol % Ir, both distances have the smallest difference, but a square base area does not occur in the cation coordination polyhedron. At the same time, the metal–metal distance (also corresponding to the lattice parameter *c*) remains the same at small doping levels and even increases above 15 mol % Ir, which means that the polyhedral centers do not approach each other, at least within the chain, raising the question of the occurrence of metal‐metal bonds as in VO_2_ (d^1^, M−M σ bond below the phase transformation temperature tetragonal to monoclinic) or MoO_2_ (d^2^, M−M σ, metallic conductivity due to the additional electron partially filling the M−O‐π* band;^[27]^ see discussion below).

### Electrical conductivity measurements

In addition to the X‐ray diffraction studies, electrical conductivity measurements were performed on the powders using a 2‐point method. Since the synthesized samples could not be pressed into tablets and no auxiliary materials were to be used for pelletizing, which could have led to reduction effects during burnout, the measurements were carried out in a modified heatable piston press with displacement transducer at different pressures at a temperature of *T*=30 °C directly on the powders, which had previously been dried again at *T*=200 °C.

**Figure 9:**
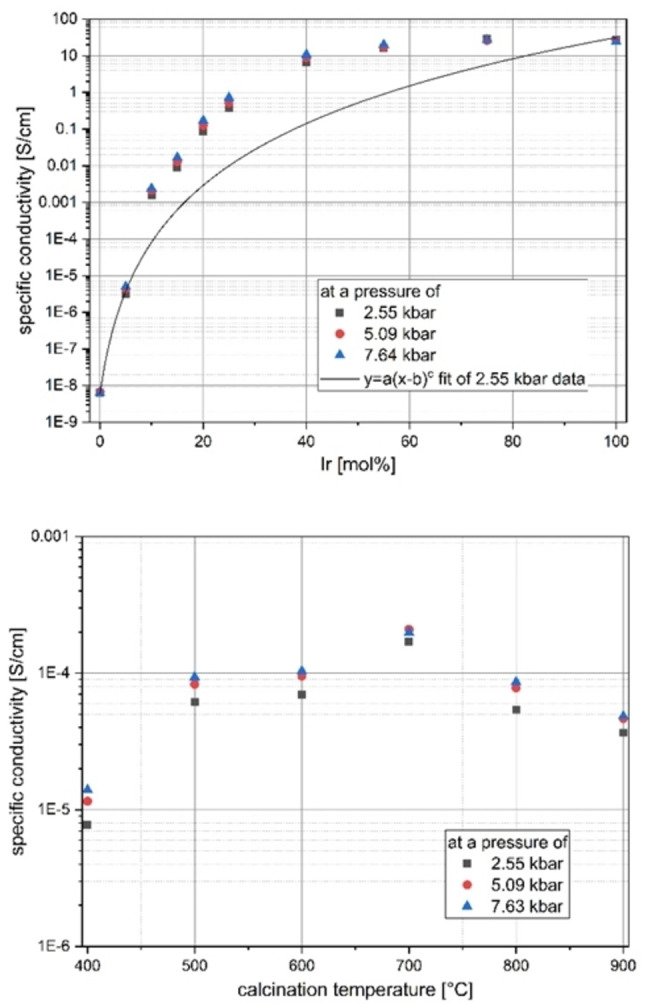
Logarithmic representations of the specific conductivities of powder samples according to the 2‐point method of the doping series Ir_x_Ti_1−x_O_2_ prepared via a modified Pechini sol‐gel route after calcination at *T*=400 °C at different pressures of the piston press (top) as well as of the calcination series of Ir_0.05_Ti_0.95_O_2_ prepared via a modified Pechini sol‐gel route after calcination at different temperatures (bottom). Explanation of the fits in the text.

Figure 9 top shows the results of the conductivity measurements of the two synthesis series (see also Table SI7).

If the percolation theory mentioned in the introduction were to apply to the TiO_2_−IrO_2_ system, that is, the samples of different composition represent a physical mixture of an electrical conductor (metal M) and an insulator, it would be expected that the specific DC conductivity σ_DC_ would increase according to Ref. [30], exceeding a percolation threshold p_C_ according to a power law with an exponent t of approximately 2.0 in the three‐dimensional percolation case σ_DC_∼σ_M_ ⋅ (p_M_−p_C_)^t^ for p_C_<p_M_ with σ_M_, the specific conductivity of the metal, and p_M_, the probability of the statistical occupation of an atomic position in the metal, that is, here the Ir doping level.

Fitting the conductivity measurement data at a pressure of *p*=2.55 kbar with the function y=a ⋅ (x−b)^c^ (black curve in Figure 9 top, Table SI7) gives only very poor agreement with the measurement data (R^2^=0.437) at an exponent c=6.2(7). Also, no usual percolation threshold can be seen at 33 mol % Ir, but the increase starts directly at 0 mol % Ir. This finding is in contrast to the results of Oakton et al.,^[31]^ who found for a series of mixed metal salt solutions of IrO_2_ and TiO_2_, prepared by a convenient one‐pot method after drying and calcination, conductivity data following the percolation model. For their samples, HAADF‐STEM imaging revealed oxide mixtures of IrO_2_ and TiO_2_, verifying the assumption of a percolation transport mechanism. The increase in conductivity in our data is extremely steep even in logarithmic representation. If the specific conductivity of pure TiO_2_ is assumed to be below 10^−8^ S/cm, it increases to more than 1 mS/cm already at 10 mol % Ir content and asymptotically approaches the values of pure IrO_2_ with about 20 S/cm for high doping contents (literature value: 30.1 S/cm^[32]^). The question arises as to the explanation for the observed behavior.

It should be noted that at higher calcination temperatures for the sample Ir_0.05_Ti_0.95_O_2_, the specific conductivity initially increases with temperature due to higher crystallinity, but decreases again above T=700 °C most probably due to segregation effects (see Figure 9 bottom, Table SI8). The overall conductivity changes with calcination temperature of this sample remain within one decade.

### Structural classification

In the series of transition metal dioxides from TiO_2_ to PtO_2_, with the exception of ZrO_2_ and HfO_2_, the rutile structure type and its distortion variants (PtO_2_: CaCl_2_ structure type) are realized throughout due to the formation of metal–metal bonds, although some of the dioxides such as MnO_2_ also crystallize in other polymorphic variants.^[33]^ The monoclinic distortions in VO_2_ (LT modification, semiconducting) and MoO_2_ (metallic) are caused by Peierls distortions of the tetragonal rutile structure, so that the metal‐metal distances alternate along the c axis. With the further occupation of M−M σ‐ and π‐bonding electron states, M−O π*‐antibonding states in RuO_2_, OsO_2_, and IrO_2_ are also occupied and these crystallize back to the undistorted rutile structure type.

**Figure 10:**
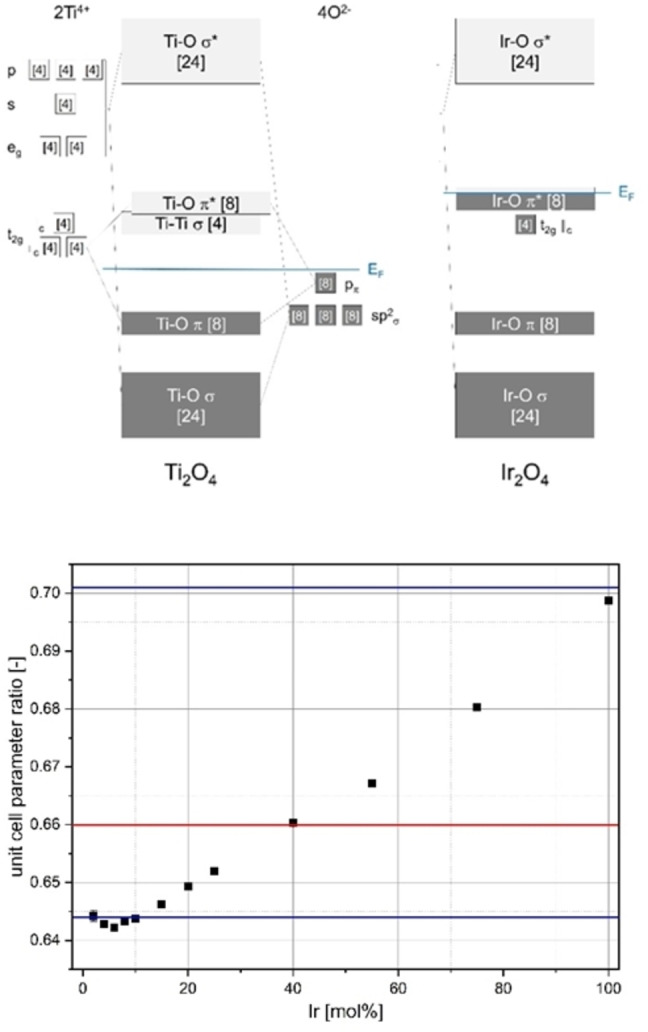
Top: schematic 1‐electron energy diagrams of Ti_2_O_4_ and Ir_2_O_4_ according to Ref. [29] with maximum occupation number in square brackets; bottom: *c*/*a* ratios of the lattice parameters of the rutile phase of samples of the Ir_x_Ti_1−x_O_2_ doping series prepared via a modified Pechini sol‐gel route after calcination at *T*=400 °C with reference values of the literature for TiO_2_ and IrO_2_ (blue) and the ideal ratio according to Pauling (red).^[34]^

Figure 10 (top) shows a simplified representation of the 1‐electron energy diagrams of TiO_2_ and IrO_2_; TiO_2_ is a semiconductor since Ti^4+^ has no occupied d states.^[27]^ The changes in the metal–metal bonds can also be seen in the *c*/*a* ratios of the lattice parameters: TiO_2_ and VO_2_ exhibit a *c*/*a*=0.644, which is smaller than the ideal ratio *c*/*a* ∼0.66 derived by Pauling^[34]^ assuming six equal M−O distances. If all metal–metal bonding and antibonding states as well as partially the M−O π*‐antibonding states are occupied as in IrO_2_, the result is a *c*/*a*=0.701 (see also Table SI1 for lattice parameters) with metal‐like conductivity. In addition to these “limits”, Figure 10 (bottom) shows the evolution of the *c*/*a* ratio in the TiO_2_−IrO_2_ doping series. It can be seen that the *c*/*a* ratio changes almost linearly from one limit to the other.

In the TiO_2_−IrO_2_ doping series, there is a continuous increase in the occupation number in the M−M non‐binding and M−O π* states from 0 to 10 electrons, relative to the M_2_O_4_ unit, from TiO_2_ to IrO_2_. This explains the observed steep increase in specific conductance values. A run of distorted structural variants of the rutile as in VO_2_ or MoO_2_ could not be determined from the X‐ray diffraction data, since the attempts of refinements of structural models with monoclinic symmetry (space group *P*2_1_ */c*) do not yield (significant) improvements of the quality factors.

## Conclusions

A complete solid solution series was set up for the TiO_2_−IrO_2_ mixed oxide series, with solid solution formation extending not only to the rutile but also to the anatase structure type. For both structure types, changes in the lattice parameters of the tetragonal unit cells were observed as a function of the doping level, although the anatase structure type could be clearly detected in the powder diffraction data only up to an Ir content of 7 mol %. At higher Ir dopant contents, only a rutile solid solution phase was found. However, possible local inhomogeneities in the Ti/Ir ratio leading to additional reflection broadening in the X‐ray diffraction data could not be completely excluded. Nevertheless, the refined Ir content follows the nominal Ir content quite well, so that larger amorphous fractions were considered unlikely. Measurements of electrical conductivity using a 2‐point method on powders showed an extremely steep increase in conductivity even at small Ir doping levels over a total of 9 decades until reaching metallic conductivity for IrO_2_ at about 20 S/cm. For technical applications as electrocatalysts, doping levels as low as 10–15 mol % Ir could be of interest, at which specific conductivities of up to 0.01 S/cm can be achieved. The observed conductivities and their dependence on the doping level do not indicate a percolation model as explanation, but a band‐like character for the additional valence electrons introduced into Ti by Ir. The linear increase of the *c*/*a* ratio also indicates a continuous filling of M−M bonding or non‐bonding states and M−O π*‐antibonding states and thus a metallic character in the TiO_2_−IrO_2_ solid solution system.

## Experimental section

In order to develop the synthesis route presented here, many preliminary experiments and investigations were carried out. These cannot be presented here in full, but justify the synthesis route, which ultimately turns out to be the most optimal, and the procedural principle for the experiments. The synthesis route is based on a sol‐gel route developed by Pechini.^[35]^ It is an extension of the original ethylene glycol route,^[36]^ a polymer complex route. The scheme in Table SI9 shows the synthesis approach for the doping series in general form. During the preparation of the synthesis steps, it was found that the sequence of chemical addition determines the gelation. Dried residues that become a clear and glassy splinter are considered “properly” gelled products. For example, ethylene glycol may be added only after the titanium precursor has stabilized. If aggregations, flocculation or sedimentation occur during gelation, which may not necessarily be reversible, it must be assumed that the formation of a mixed oxide becomes unlikely due to this segregation of the components. Mixed oxide formation is always based on the distribution of the components contained at the atomic level in the solution and during the gelation process.

**Synthesis protocol of Ir_x_Ti_1−x_O_2_ (0≤x≤1)**: 0.2 M citric acid solution (in 1‐propanol: HSL 99 %, citric acid: Grüssing GmbH, 99 %, batch number: 1272) was placed in a 50 mL rolled rim glass vial. The amount depends on the degree of doping and ranges from 5 to 21 mL for a batch size of 1 mmol oxide. The appropriate amount of titanium(IV)‐*iso*‐propoxide (Sigma–Aldrich, 97 %) was then added under the exposure of nitrogen gas (Air Liquide, nitrogen 5.0). The calculation is based on 1 mmol of oxide. Thus, for a nominal doping level of 5 mol % Ir (x=0.05 or 5 mol %), 950 μmol titanium precursor and 50 μmol iridium precursor were used. The density of the liquid titanium precursor was considered to be ρ=0.96 g/cm^3^. The size of the batch had an influence on the course of the syntheses. Therefore, at higher doping levels (from x=11 mol %), several batches were performed and combined, sometimes with only 0.06 mmol batch size. Additional combined multiple batches were required for sufficient sample volume for powder X‐ray diffraction characterization. The β‐diketone acetylacetone (Roth, 98 %) was added to stabilize the titanium precursor.[^37^, ^38^, ^39^, ^40^, ^41^] The presence of acetylacetone has a positive effect on the gelation process as it can stabilize hydrolysis‐sensitive precursors by complexation according to Refs. [14,42] and [43]. The result is a yellow solution that can be stored even with air ingress. The volume of the solutions depends on the degree of doping and was between 2.5 and 10.5 mL. Ethylene glycol (Grüssing GmbH, 99 %, batch number: 2170) was then added. The polymer formers citric acid and ethylene glycol were always added in a volume ratio of 1 : 1. Polycondensation of ethylene glycol with citric acid or oxidized as oxalic acid can form a branched polyester to whose functional groups the metal ions can attach. This non‐classical sol‐gel route is referred to in the literature as the polymerized complex method (PCM).^[44]^ The organic polymer penetrates an inorganic solid network,^[45]^ preventing the formation of large condensed aggregates of the metal compounds used. Thus, a finer distribution of metals in binary metal oxides can be achieved. According to Ref. [46], the formation of adducts or esters from citric acid (CA) or its more or less deprotonated forms, ethylene glycol (EG) and the metal ions of a binary system (yttrium‐titanium) was demonstrated. The authors used NMR and FTIR analyses to detect the intermediates. After preparation, complexes of the following form are formed: (EG)‐(CA)‐Ti^4+^‐Y^3+^. The mixture was then further mixed with the iridium precursor solution (iridium acetylacetonate dissolved in 1 : 1 acetylacetone:methanol, concentration 0.03 mol/L; iridium precursor: Heraeus, precious metal content: 39.3 %, batch number: 10313; methanol: VWR, HPLC grade). The amount of acetonitrile added varied from 4.5 to 16.5 mL per 1 mmol batch size, depending on the doping level. It should be noted that due to the increasing amount of iridium precursor solution with increasing doping level, more solvent must also be available for solubilization and stabilization of the metal compounds. Overall, it can be seen that significantly more solvent is required at higher doping levels to prevent precipitation during gelation. This is due to the poor solubility of the iridium precursor. A few drops of concentrated nitric acid (HSL, purest) were then added and a gelation temperature of *T*=100 °C was set on the heated vortexer. In preliminary experiments, the formation of metallic precipitates after calcination was observed more frequently, which could be minimized by adding larger amounts of oxidizing acid. In the preparation of pure iridium dioxide, the addition of hydrogen peroxide prior to calcination is also suitable to oxidize the precursor from the trivalent to the tetravalent stage. Syntheses were performed in 50 mL rolled‐rim glass vials with a height of 10 cm. The preparation volume was limited to allow the mixture to gel at *T*=100 °C similar to reflux. The partially recondensed solvents flowed back along the wall of the vial so that sufficient solvent was present over the entire period to prevent precipitation. The preparations took between one and two weeks to form dry, glassy, splintered and clear gels. Especially from about 15 mol % Ir, there was no fragmentation of the gels. Here, dark brown polymer blocks were obtained. The mass of the dried gels was about 0.5 g. Calcination up to 400 °C was then carried out in a muffle furnace (thermicon‐P, Heraeus Instruments). Homogeneous brown powders were obtained at low doping levels. Above a doping level of 4.5 mol % Ir, the powders are black.

Details of the syntheses protocols: Ir_0.05_Ti_0.95_O_2_ (x=0.05 or 5 mol%) as described above with the following amounts: 950 μmol titanium precursor and 50 μmol iridium precursor; 2.9 mL acetylacetone, 5.8 mL ethylene glycol, 5.8 mL citric acid solution in 1‐propanol of 0.20 mol/L concentration, 5.1 mL acetonitrile; Ir_0.40_Ti_0.60_O_2_ (x=0.40 or 40 mol%) as described above with the following amounts: 600 μmol titanium precursor and 400 μmol iridium precursor; 5.7 mL acetylacetone, 11.4 mL ethylene glycol, 11.4 mL citric acid solution in 1‐propanol of 0.20 mol/L concentration, 9.3 mL acetonitrile, 1 mL conc. nitric acid. Note that the synthesis strategy has not been driven by mechanistic considerations but simply empirically: in a large number of experiments the synthesis conditions have been evaluated by registering the precipitation of unwanted products during the gelling process as we identified them as the pure oxides and not mixed oxides. Above we specified the exact amounts of all components for two doping levels, 5 and 40 mol% Ir. Thus, all other compositions can be synthesized from these data by linear inter‐/extrapolation of these values.

**Calcination**: Table SI10 shows the calcination program used for the doping series. A temperature program with low heating rates was chosen to avoid disproportionation of the iridium organics in iridium dioxide and metallic iridium due to insufficient oxygen access. The program is based on findings from decomposition studies,^[47]^ which were confirmed by TGA/DSC investigations. For the sol‐gel method used here, the calcination method represents a very important point. It was carried out in a muffle furnace under oxidizing conditions, and stable products can be obtained under ambient conditions. The final temperature of calcination was limited to *T*=400 °C to prevent segregation, which would probably occur at higher temperatures. However, this resulted in products with a relatively small crystallite size.

**Characterization**: powder X‐ray diffraction with Rietveld refinement PXRD/ *Topas*: Bruker D8 diffractometer with Co fine focus tube (λ_Kα_=1,79021 Å, Θ‐Θ geometry, VDS, Lynxeye detector); phase identification according to CIF files listed in Table SI11, *Topas* program version 4.2, evaluation method for the Rietveld refinement;[^20^, ^48^, ^49^] simultaneous thermal analysis measurements TGA/DSC for Ir_0_Ti_1_O_x_ and Ir_0.05_Ti_0.95_O_x_: Mettler Toledo TGA/DSC 2 Star^e^ system, degradation of the organics completed at Ir_0_Ti_1_O_x_: T=470 °C, at Ir_0.05_Ti_0.95_O_x_: 380 °C.

**Conductivity measurements according to the 2‐point method**: A laboratory press from Paul‐Otto Weber GmbH was used for the specific conductivity measurements. The PW 10 E‐PRESSYS model used allows the setting of pressing forces in the range of 10–130 kN with a compact hydraulic unit. With proportional pressure control valve technology and pressure sensors, it is possible to run pressure‐programmed ramps. The LS 1679 incremental length measuring system with integrated roller guide is installed in the laboratory press. With this device it is possible to measure the change in distance of the press plate position, but not position control. According to the manufacturer, the accuracy of the system is ±10 μm. The device registers the position of the lifting arm when it is switched on. This allows coating thicknesses to be determined as a subtraction between a measurement with a sample and a blank measurement of the hot pressing tool. The Model 10 HS hot pressing tool was used, which has a pressing plate bore diameter of 13 mm. This allows an electrically insulating sleeve made of PEEK or Teflon to be placed around the 10 mm press pistons to prevent a short circuit between the steel pistons and the die plate. However, this makes the temperature setting sluggish due to the poor thermal conductivity of the material. Measurements were performed at *T*=30 °C on selected samples of the Ir_x_Ti_1−x_O_2_ system. Keithley's Source Meter 2400 was used to determine electrical resistance, with the direction of current in the measurements inverted for every other data point. Synchronous data recording of all press parameters, temperature at the pressing tool, and source meter parameters was implemented using a *LabView* program. Details of the calibration and reference measurements are described in a separate publication.

## Supporting Information Summary

Additional information is available as Supporting Information, detailing PXRD data as reflection position shifts and intensity changes with doping level and Rietveld refinement parameters as changes in positional parameters and site occupation factors in form of graphical representations. Included are also a comparison of the refinement of the X‐ray diffraction pattern observed with two alternative models and parameter changes with different calcination temperatures. Finally all Rietveld refinement parameters and conductivity measurement data are included in detail in tables.

## Conflict of interest

The authors declare no conflict of interest.

1

## Supporting information

As a service to our authors and readers, this journal provides supporting information supplied by the authors. Such materials are peer reviewed and may be re‐organized for online delivery, but are not copy‐edited or typeset. Technical support issues arising from supporting information (other than missing files) should be addressed to the authors.

Supporting Information

## Data Availability

The data that support the findings of this study are available from the corresponding author upon reasonable request.
