# Supplementary material for: Sol‐Gel‐Syntheses and Structural as well as Electrical Characterizations of Anatase‐ and Rutile‐Type Solid Solutions in the System IrO2−TiO2
Source: ChemistryOpen. 2023 Jul 19;12(7):e202300032. doi: 10.1002/open.202300032 (PMC10356930; doi:10.1002/open.202300032)
Supplement: Supplementary file 1 — Supporting Information [file OPEN-12-e202300032-s001.pdf]

ChemistryOpen

Supporting Information

Sol-Gel-Syntheses and Structural as well as Electrical Characterizations of Anatase- and Rutile-Type Solid Solutions in the System IrO_2–TiO_2

Daniel Reichert and Klaus Stöwe*

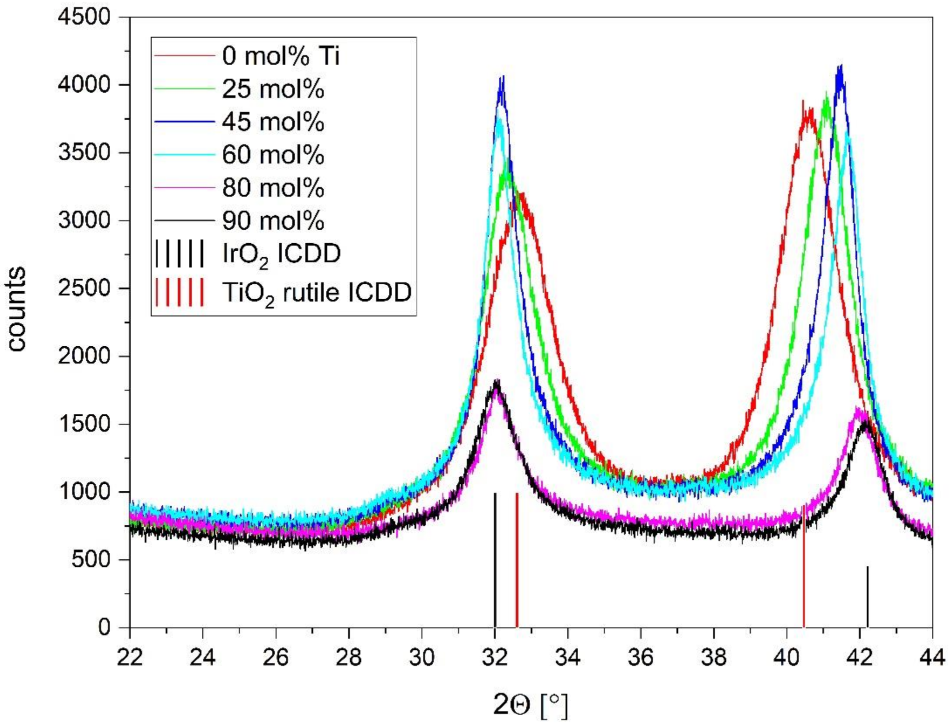

Figure SI 1. Shift of the PXRD reflection positions (110) and (011) in the rutile phases of Ir-doped TiO_2 samples with doping levels between 10 and 100% Ir synthesized via a modified Pechini sol-gel route after calcination at T = 400 °C. Vertical lines in diagram as well as in inset represent ICDD data as references (IrO_2: 88-0288 and rutile TiO_2: 73-1765)

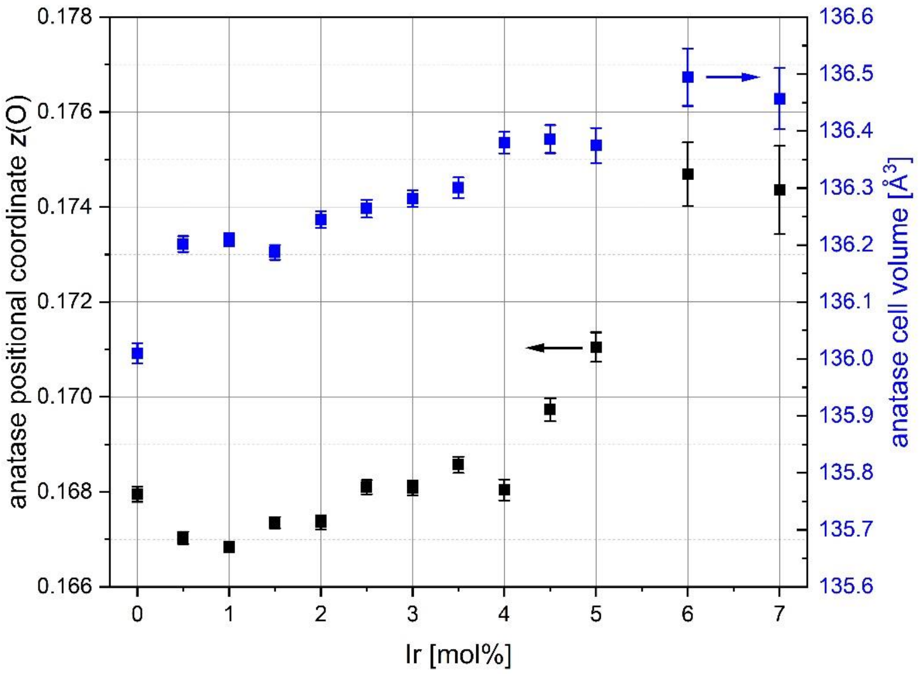

Figure SI 2. Change of the positional parameter of the oxygen atoms z and the unit cell volume in the anatase structure-type in the doping series $Ir_xTi_{1-x}O_2$ synthesized via a modified Pechini sol-gel route after calcination at *T* = 400 °C; above 7 mol% Ir the reflection intensities of the anatase phase are too weak to be evaluated quantitatively.

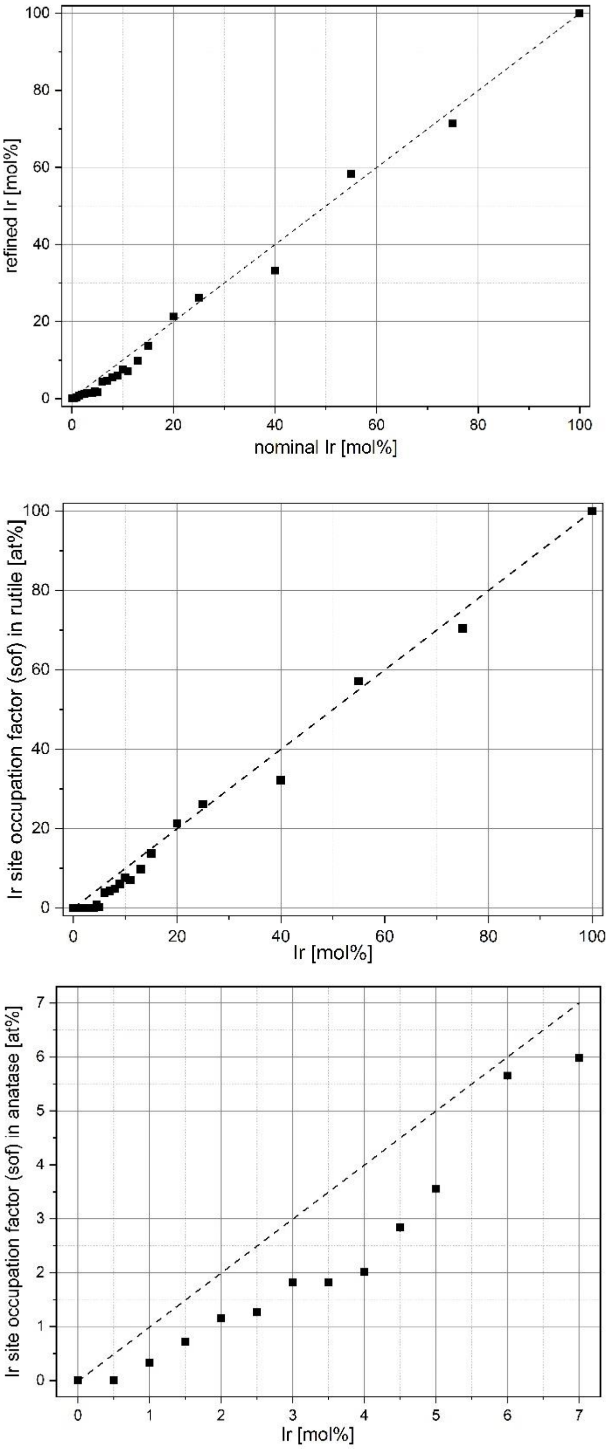

Figure SI 3. Site occupation factors of Ir of the cation position M (4a) 000 in the space group *I4₁/amd* (top) and M (2a) 000 in the space group *P4₂/mnm* (middle) in solid solutions with anatase and rutile crystal structure, respectively, of the doping series $Ir_xTi_{1-x}O_2$ synthesized via a modified Pechini sol-gel route after calcination at T = 400 °C; nominal and refined Ir content in mol% in comparison (bottom). Angel bisectors as dashed lines for eye guidance.

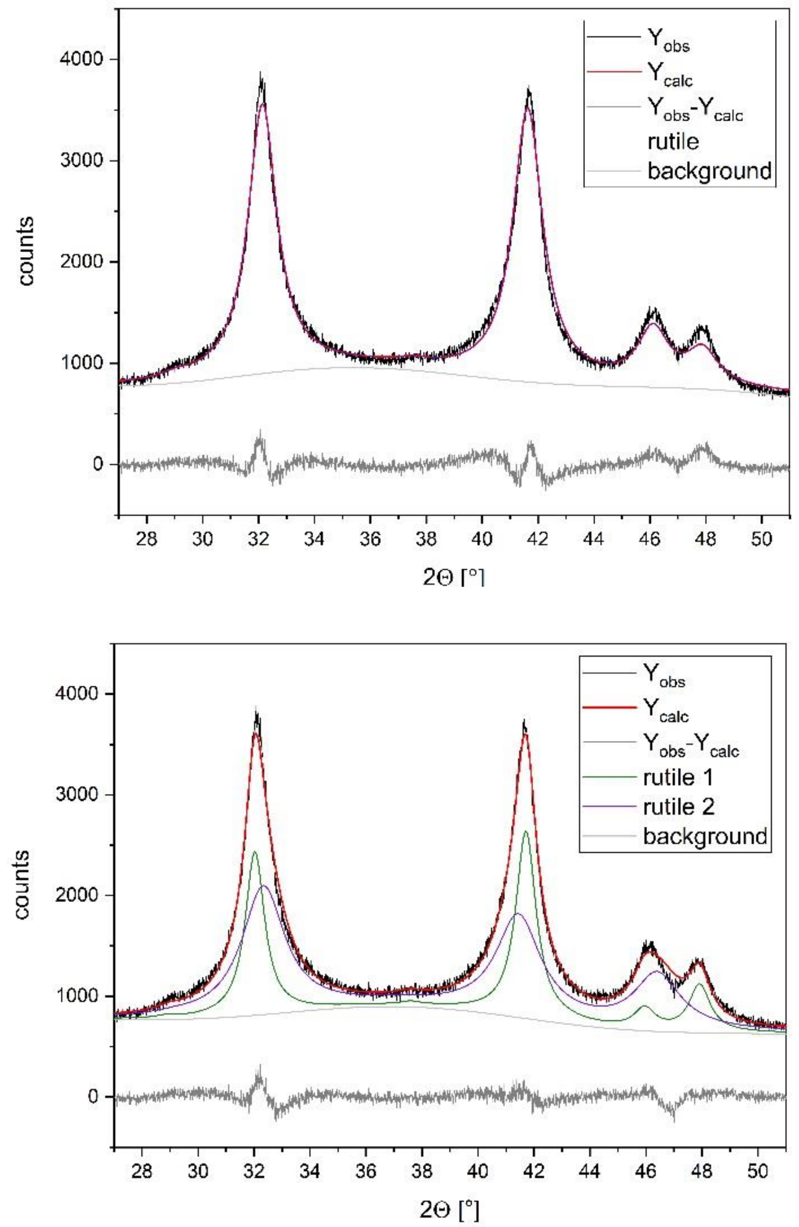

Figure SI 4. Comparison of the refinement of the X-ray diffraction pattern observed to two alternative models with one (model 1, top) and two solid solution phases in rutile structure type (model 2, bottom) refined with Topas; legend: black - PXRD measurements Y_{obs}, red - refined diffractograms Y_{ref}; green, violet - refined single phases; dark grey - difference between measurement and refined values, light grey - background; sample $Ir_{0.4}Ti_{0.6}O_2$ synthesized via a modified Pechini-Sol-Gel route after calcination at T = 400 °C.

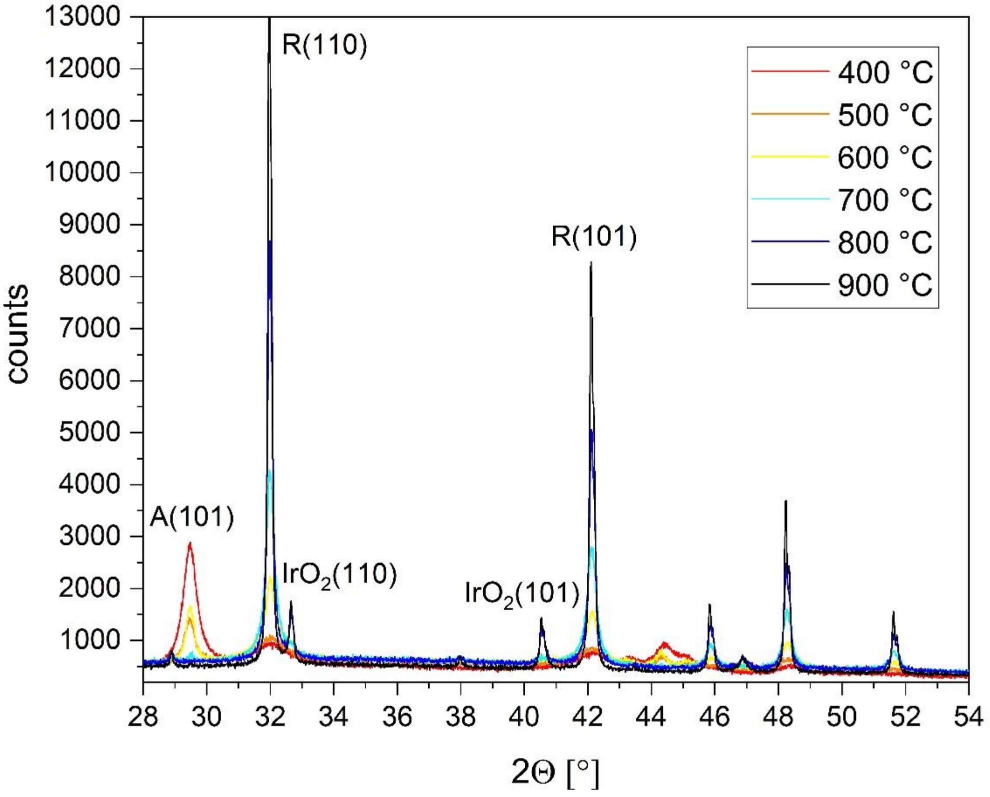

Figure SI 5. Change in PXRD intensities of some reflections of the occurring phases anatase (A), rutile (R) TiO_2 and IrO_2 for $Ir_{0.05}Ti_{0.95}O_2$ at different calcination end temperatures.

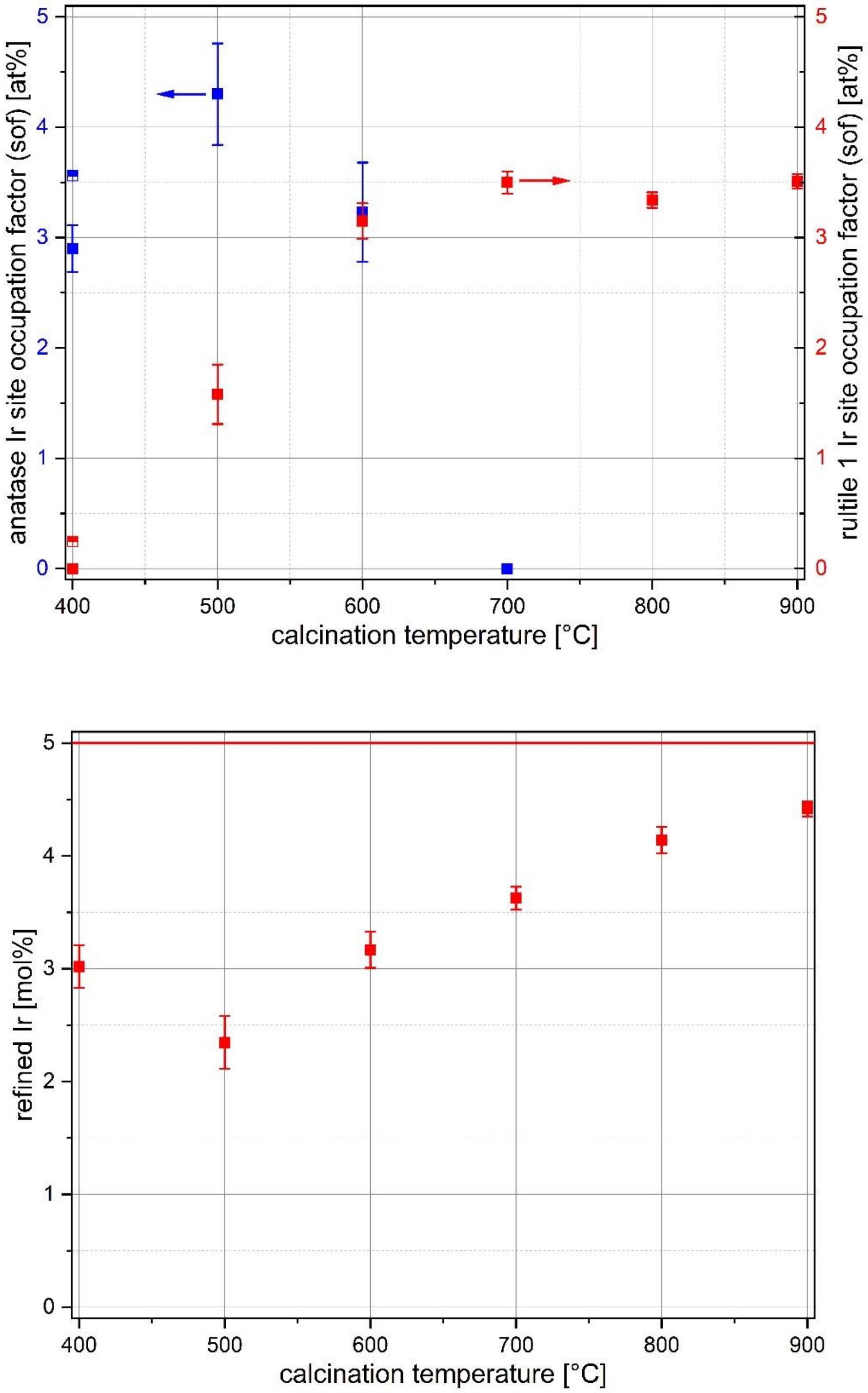

Figure SI 6. Top: Temperature dependence of Ir site occupation factor in samples of composition $Ir_{0.05}Ti_{0.95}O_2$ prepared by a modified Pechini-Sol-Gel route after calcination at different temperatures (blue: anatase, red: rutile 1 phase; semi-filled symbols at *T* = 400°C: values from doping series); bottom: refined Ir content as a function of calcination temperature.

Table SI 1: Lattice parameters as function of doping level

Doping level Ir	Phase fraction Ir	Phase fraction anatase	Phase fraction rutile	Total Ir amount refined	Total Ir amount refined	Anatase lattice parameter *a*	Error a	Anatase lattice parameter *c*	Error *c*	Rutile lattice parameter *a*	Error *a*	Rutile lattice parameter *c*	Error *c*
[mol%]	[wt%]	[wt%]	[wt%]	[wt%]	[mol%]	[Å]	[Å]	[Å]	[Å]	[Å]	[Å]	[Å]	[Å]
0	0	100	0	0	0.000	3.7852	0.0002	9.4927	0.0006				
0.5	0	100	0	0	0.000	3.7866	0.0002	9.4993	0.0005				
1	0	100	0	0.328	0.328	3.7871	0.0001	9.4973	0.0004				
1.5	0	100	0	0.718	0.718	3.7875	0.0002	9.4934	0.0004				
2	0	94.1	5.9	1.090	1.088	3.7888	0.0002	9.4912	0.0005	4.589	0.003	2.957	0.003
2.5	0	90.2	9.8	1.142	1.139	3.7897	0.0002	9.4881	0.0005	4.588	0.002	2.954	0.002
3	0	83.9	16.1	1.529	1.521	3.7904	0.0002	9.4856	0.0005	4.593	0.001	2.954	0.001
3.5	0	80.1	19.9	1.458	1.448	3.7912	0.0002	9.4828	0.0006	4.591	0.001	2.950	0.001
4	0	73.2	26.8	1.476	1.462	3.7921	0.0002	9.4838	0.0006	4.590	0.001	2.951	0.001
4.5	0	57.4	42.6	2.000	1.984	3.7934	0.0003	9.4779	0.0008	4.5923	0.0007	2.9488	0.0007
5	0	44.4	55.6	1.716	1.669	3.7941	0.0004	9.474	0.001	4.5938	0.0007	2.9468	0.0006
6	0	23.4	76.6	4.293	4.284	3.7970	0.0006	9.468	0.002	4.5954	0.0007	2.9511	0.0006
7	0	14.4	85.6	4.541	4.535	3.7969	0.0006	9.466	0.002	4.5944	0.0007	2.9515	0.0005
8	0	8.2	91.8	5.714	5.584	3.7515	0.0028	9.63	0.02	4.5937	0.0008	2.9550	0.0005
9	0	0	100	6.007	6.007					4.5940	0.0008	2.9549	0.0006
10	0	0	100	7.637	7.637					4.5917	0.0007	2.9561	0.0005
11	0	0	100	7.051	7.051					4.5908	0.0008	2.9542	0.0006
13	0	0	100	9.808	9.808					4.5886	0.0008	2.9577	0.0006

Contin. **Table SI 1**

Doping level Ir	Phase fraction Ir	Phase fraction anatase	Phase fraction rutile	Total Ir amount refined	Total Ir amount refined	Anatase lattice parameter a	Error *a*	Anatase lattice parameter *c*	Error *c*	Rutile lattice parameter *a*	Error *a*	Rutile lattice parameter *c*	Error *c*
[mol%]	[wt%]	[wt%]	[wt%]	[wt%]	[mol%]	[Å]	[Å]	[Å]	[Å]	[Å]	[Å]	[Å]	[Å]
15	0	0	100	13.700	13.700	-	-	-	-	4.5882	0.0008	2.9649	0.0006
20	0	0	100	21.270	21.270	-	-	-	-	4.5844	0.0007	2.9765	0.0006
25	0.15	0	99.85	26.281	26.238	-	-	-	-	4.5815	0.0005	2.9872	0.0005
40	2.41	0	97.59	33.814	33.263	-	-	-	-	4.5679	0.0004	3.0159	0.0004
55	3.1	0	96.9	58.459	58.258	-	-	-	-	4.5574	0.0004	3.0404	0.0003
75	3.4	0	96.6	71.416	71.362	-	-	-	-	4.5326	0.0003	3.0834	0.0003
100	2.7	0	97.3	100.000	100.000	-	-	-	-	4.4971	0.0003	3.1420	0.0003

Table SI 2 Anatase structural parameters as function of doping level

Doping level Ir	Phase fraction	Positional parameter *z*(O)	Error *z*(O)	Cell volume *V*	Error *V*	Crystallite size *L*	Error *L*	Site occupation factor *sof*(Ti)	Error *sof*(Ti)
[mol%]	[wt%]	[-]	[-]	[Å^3]	[Å^3]	[nm]	[nm]	[-]	[-]
0	100	0.1680	0.0001	136.01	0.02	37.1	0.8	1	0
0.5	100	0.1670	0.0001	136.20	0.01	59.1	1.2	1	0
1	100	0.1668	0.0001	136.21	0.01	73.5	1.5	0.997	0.001
1.5	100	0.1674	0.0001	136.19	0.01	63.9	1.3	0.993	0.001
2	94.1	0.1674	0.0002	136.24	0.02	60.4	1.3	0.988	0.001
2.5	90.2	0.1681	0.0002	136.26	0.02	65	1.6	0.987	0.001
3	83.9	0.1681	0.0002	136.28	0.02	61	1.3	0.982	0.001
3.5	80.1	0.1686	0.0002	136.30	0.02	58.3	1.5	0.982	0.001
4	73.2	0.1680	0.0002	136.38	0.02	67.4	2.3	0.980	0.002
4.5	57.4	0.1697	0.0002	136.39	0.03	52.9	1.5	0.972	0.002
5	44.4	0.1711	0.0003	136.37	0.03	58.8	2.3	0.964	0.003
6	23.4	0.1747	0.0007	136.49	0.05	36.9	1.8	0.944	0.006
7	14.4	0.1744	0.0009	136.46	0.05	38.1	2.1	0.940	0.008
8	8.2	0.221	0.006	135.6	0.4	5.3	0.5	0.84	0.05

Table SI 3: Rutile structural parameters as function of doping level

Doping level Ir	Phase fraction	Positional parameter *x*(O)	Error *x*(O)	Cell volume *V*	Error *V*	Crystallite size *L*	Error *L*	Site occupation factor *sof*(Ti)	Error *sof*(Ti)
[mol%]	[wt%]	[-]	[-]	[Å^3]	[Å^3]	[nm]	[nm]	[-]	[-]
2	5.9	0.296	0.004	62.27	0.09	10.1	1.5	1	0
2.5	9.8	0.294	0.003	62.19	0.06	9.9	0.9	1	0
3	16.1	0.292	0.002	62.31	0.04	8.2	0.5	1	0
3.5	19.9	0.290	0.002	62.19	0.04	8.2	0.4	1	0
4	26.8	0.293	0.002	62.18	0.04	7.5	0.2	1	0
4.5	42.6	0.290	0.001	62.19	0.02	7.4	0.1	0.991	0.003
5	55.6	0.291	0.001	62.19	0.02	7.3	0.1	0.998	0.003
6	76.6	0.2905	0.0005	62.32	0.02	7.9	0.1	0.961	0.003
7	85.6	0.2903	0.0005	62.30	0.02	7.7	0.1	0.957	0.002
8	91.8	0.2899	0.0006	62.36	0.02	7.9	0.1	0.952	0.003
9	100	0.2893	0.0006	62.36	0.03	7.7	0.1	0.940	0.003
10	100	0.2877	0.0005	62.33	0.02	7.6	0.1	0.924	0.003
11	100	0.2861	0.0006	62.26	0.03	7.1	0.1	0.930	0.003
13	100	0.2852	0.0006	62.28	0.03	7.1	0.1	0.902	0.003
15	100	0.2851	0.0007	62.42	0.02	7.5	0.1	0.863	0.004
20	100	0.2862	0.0007	62.56	0.02	7.7	0.1	0.787	0.005
25	99.85	0.2880	0.0007	62.70	0.02	8	0.1	0.738	0.006
40	97.59	0.2897	0.0006	62.93	0.01	8	0.1	0.678	0.005
55	96.9	0.2891	0.0007	63.15	0.01	8.7	0.1	0.429	0.012
75	96.6	0.2878	0.0008	63.35	0.01	5.7	0.1	0.296	0.018
100	97.3	0.2849	0.0009	63.54	0.01	4.6	0.1	0	0

Table SI 4. Comparison of refined structural parameters of model 1 and model 2 for a sample of composition $Ir_{0.4}Ti_{0.6}O_2$ synthesized via a modified Pechini-Sol-Gel route after calcination at *T* = 400 °C.

**model 1: $Ir_{0.32}Ti_{0.68}O_2$, R_{wp} = 5.258**					
formula ref.	*a* [Å]	*c* [Å]	*x*(O)	*sof*(Ir)	fraction [%]
$Ir_{0.32}Ti_{0.68}O_2$	4.5678(4)	3.0159(4)	0.2897(6)	0.322(5)	100
**model 2: $Ir_{0.26}Ti_{0.74}O_2$, R_{wp} = 4.342**					
formula ref.	*a* [Å]	c [Å]	*x*(O)	*sof*(Ir)	fraction [%]
$Ir_{0.12}Ti_{0.88}O_2$	4.5809(4)	3.0026(4)	0.2730(9)	0.116(6)	80(1)
$Ir_{0.81}Ti_{0.19}O_2$	4.5394(9)	3.0419(9)	0.395(3)	0.81(5)	20(3)
**references**					
formula	*a* [Å]	*c* [Å]	*x*(O)	*sof*(Ir)	ref.
TiO_2	4.594(3)	2.959(2)	0.306(1)	0	[32]
IrO_2	4.5051(3)	3.1586(2)	0.3077(3)	1	[31]

$$R_{wp} = \sqrt{\frac{\sum w_m (Y_{obs,m} - Y_{calc,m})^2}{\sum w_m Y_{obs,m}^2}}$$

It can therefore be seen that this sample could also be interpreted as a mixture of two rutile solid solutions (a mixed crystal rich in iridium and a mixed crystal poor in iridium), but the even greater deviation of the refined composition from the nominal one and the unusually large *x* parameters for the oxygen ions in the second rutile phase is to be noted in model 2. Furthermore, the lattice parameters obtained from the refinement of model 2 do not fit into the relationships shown in Figure 4 (main text). The reflection profile refinement of the two most intensive reflections of the rutile phase is improved by the larger number of parameters and thus the quality of the refinement increases recognizably by the smaller R_{wp} value.

Table SI 5: Temperature series of $Ir_{0.05}Ti_{0.95}O_2$

a) Anatase:

Annealing temperature	Phase fraction	Lattice parameter *a*	Error a	Lattice parameter *c*	Error c	Positional parameter *z*(O)	Error *z*(O)	Cell volume *V*	Error *V*	Crystallite size *L*	Error *L*	Site occupation factor *sof*(Ir)	Error *sof*(Ir)
[°C]	[wt%]	[Å]	[Å]	[Å]	[Å]	[-]	[-]	[Å^3]	[Å^3]	[nm]	[nm]	[-]	[-]
400*	44.4	3.7941	0.0004	9.474	0.001	0.1711	0.0003	136.38	0.03	59	2	0.036	0.003
400	52.6	3.7939	0.0004	9.473	0.001	0.1702	0.0003	136.35	0.03	34	1	0.029	0.002
500	29.1	3.7927	0.0005	9.487	0.001	0.1722	0.0005	136.47	0.04	47	2	0.043	0.005
600	22.4	3.7896	0.0002	9.501	0.001	0.1728	0.0006	136.44	0.02	68	4	0.032	0.005
700	1.12	3.7798	0.0008	9.546	0.005	0.200	0.006	136.4	0.1	94	38	0	0
800	0												
900	0												

b) Rutile 1

Annealing temperature	Phase fraction	Lattice parameter *a*	Error a	Lattice parameter *c*	Error c	Positional parameter *x*(O)	Error *x*(O)	Cell volume *V*	Error *V*	Crystallite size *L*	Error *L*	Site occupation factor *sof*(Ir)	Error *sof*(Ir)
[°C]	[wt%]	[Å]	[Å]	[Å]	[Å]	[-]	[-]	[Å^3]	[Å^3]	[nm]	[nm]	[-]	[-]
400*	55.6	4.5938	0.0007	2.9468	0.0006	0.2913	0.0007	62.19	0.02	7.3	0.1	0.016	0.003
400	47.4	4.5937	0.0006	2.9545	0.0006	0.292	0.006	62.35	0.02	7.3	0.1	0.032	0.002
500	70.9	4.5940	0.0006	2.9585	0.0005	0.2989	0.0006	62.44	0.02	10.1	0.1	0.035	0.001
600	77.6	4.5935	0.0003	2.9629	0.0002	0.3045	0.0003	62.52	0.01	20.7	0.1	0.033	0.001
700	98.49	4.5929	0.0001	2.9637	0.0001	0.3052	0.0002	62.520	0.003	39.8	0.2	0.035	0.001
800	98.14	4.5997	0.00004	2.96303	0.00003	0.3056	0.0002	62.510	0.001	104.7	0.5	0.016	0.003
900	97.9	4.59297	0.00002	2.96293	0.00001	0.3051	0.0001	62.510	0.005	261.3	2.1	0.032	0.002

*see doping series Table Si 1

Contin. **Table SI 5:** Temperature series of $Ir_{0.05}Ti_{0.95}O_2$

Rutile 2 = IrO_2

Annealing temperature	Phase fraction	Lattice parameter *a*	Error a	Lattice parameter *c*	Error c	Positional parameter *x*(O)	Error *x*(O)	Cell volume *V*	Error *V*	Crystallite size *L*	Error *L*	Site occupation factor *sof*(Ir)	Error *sof*(Ir)
[°C]	[wt%]	[Å]	[Å]	[Å]	[Å]	[-]	[-]	[Å^3]	[Å^3]	[nm]	[nm]	[-]	[-]
400													
400													
500													
600													
700	0.39	4.5013	0.0009	3.151	0.001	0.44	0.02	63.85	0.03	56	8	1	0
800	1.86	4.4992	0.0001	3.1524	0.0001	0.322	0.003	63.820	0.004	108	3	1	0
900	2.10	4.4993	0.0001	3.1512	0.0001	0.323	0.003	63.800	0.003	123	3	1	0

Table SI 6: Selected interatomic distances of rutile type solid solution $Ir_xTi_{1-x}O_2$ as function of doping level

Doping level Ir	d(axial)	Error d(axial)	d(equatorial)	Error d(equi)	Lattice para-meter *c*	Error *c*	Shared edge	Error shared edge
[mol%]	[pm]	[pm]	[pm]	[pm]	[pm]	[pm]	[pm]	[pm]
2	191	3	199	1	295.7	0.3	267	3
4	189.9	0.8	200.0	0.5	295.3	0.1	269.7	1.1
6	188.8	0.3	200.8	0.2	295.11	0.06	272.2	0.4
8	188.5	0.3	201.0	0.2	295.50	0.05	272.7	0.4
10	186.8	0.3	202.1	0.2	295.61	0.05	275.7	0.4
15	185.0	0.3	203.5	0.3	296.49	0.06	278.8	0.5
20	185.6	0.3	203.4	0.3	297.65	0.06	277.1	0.5
25	186.6	0.3	202.9	0.3	298.72	0.05	274.7	0.5
40	187.2	0.3	203.0	0.2	301.60	0.04	271.7	0.4
55	186.3	0.4	204.0	0.3	304.04	0.03	272.0	0.5
75	184.5	0.4	205.6	0.3	308.34	0.03	272.1	0.5
100	181.2	0.4	208.3	0.3	314.20	0.03	273.6	0.6

Table SI 7 Specific conductivities of solid solutions $Ir_xTi_{1-x}O_2$ as function of doping level

Doping level Ir	Specific conductivity σ @ 2.55 kbar	Error σ @ 2.55 kbar	Specific conductivity σ @ 5.09 kbar	Error σ @ 5.09 kbar	Specific conductivity σ @ 7.64 kbar	Error σ @ 7.64 kbar
[mol%]	[S/cm]	[S/cm]	[S/cm]	[S/cm]	[S/cm]	[S/cm]
0	6.253E-09	1.684E-12	6.749E-09	2.551E-12	6.385E-09	2.724E-12
5	3.127E-06	1.355E-10	4.424E-06	6.808E-10	4.960E-06	1.807E-09
10	1.582E-03	7.539E-08	2.119E-03	2.882E-07	2.386E-03	7.225E-07
15	8.775E-03	2.744E-07	1.283E-02	1.886E-06	1.669E-02	2.136E-06
20	8.840E-02	1.504E-06	1.260E-01	2.242E-05	1.710E-01	3.420E-06
25	3.819E-01	8.398E-06	5.242E-01	1.019E-04	7.148E-01	5.070E-05
40	6.694E+00	1.467E-03	8.689E+00	6.445E-03	1.081E+01	6.066E-03
55	1.611E+01	6.865E-03	1.737E+01	3.379E-02	1.995E+01	2.262E-02
75	2.849E+01	8.003E-03	2.608E+01	9.497E-02	2.703E+01	3.577E-02
100	2.764E+01	6.400E-03	2.602E+01	5.063E-03	2.461E+01	4.109E-02

Table SI 8: Specific conductivities of solid solution $Ir_{0.05}Ti_{0.95}O_2$ as function of temperature

Annealing temperature	Specific conductivity σ @ 2.55 kbar	Error σ @ 2.55 kbar	Specific conductivity σ @ 5.09 kbar	Error σ @ 5.09 kbar	Specific conductivity σ @ 7.64 kbar	Error σ @ 7.64 kbar
[°C]	[S/cm]	[S/cm]	[S/cm]	[S/cm]	[S/cm]	[S/cm]
400	7.771E-06	5.106E-10	1.154E-05	4.721E-09	1.398E-05	7.822E-09
500	6.120E-05	1.735E-09	8.283E-05	1.694E-08	9.339E-05	4.284E-08
600	6.961E-05	2.625E-09	9.515E-05	2.429E-08	1.036E-04	6.410E-08
700	1.709E-04	8.236E-09	2.088E-04	9.913E-08	1.980E-04	2.653E-07
800	5.414E-05	1.983E-09	7.787E-05	2.089E-08	8.605E-05	5.929E-08
900	3.657E-05	2.113E-09	4.650E-05	1.450E-08	4.826E-05	3.553E-08

Table SI 9. Synthesis recipe for doping series.

sequence	substance	effect
1.	citric acid	complexing agent, polymer formation
2.	titanium(IV) isopropoxide	precursor
3.	acetylacetone (+methanol)	ß-diketone stabilisation (+solvent)
4.	ethylene glycol	polymer formation
5.	iridium(III) acetylacetonate	precursor
6.	acetonitrile	solvent
7.	conc. nitric acid	catalyst for polymer formation

Table SI 10. Calcination program of muffle oven.

T [°C]	25	110	110	250	250	400	400
t [min]	-	45	180	140	210	300	240
T/t [K/min]	-	2	-	1	-	0,5	-

Table SI 11. Crystallographic Information Files (CIF) used for phase identification.

	ICSD[1]/AMCSD[2]	publication year
TiO_2 anatase	154601[1]	2007
TiO_2 rutile	9161[1]	1980, update: 2006
IrO_2 rutile	0019243[2]	1997
Ir	640730[1]	2008, update: 2009
